# Association of sleep disturbance during breast cancer treatment and left ventricular ejection fraction decline: a prospective cohort study

**DOI:** 10.1186/s40959-026-00462-6

**Published:** 2026-02-24

**Authors:** Warren Szewczyk, Kerryn W. Reding, Ralph B. D’Agostino, Richard K. Cheng, Oxana Palesh, Kristine Olson, Amy Ladd, Kathryn E. Weaver, Bonnie Ky, W. Gregory Hundley

**Affiliations:** 1 University of Washington School of Nursing, Box 357260, 1959 NE Pacific St., Seattle, WA 98195 USA; 2 Wake Forest University School of Medicine, Winston-Salem, USA; 3 University of Washington School of Medicine, Seattle, USA; 4 Virginia Commonwealth University School of Medicine, Richmond, USA; 5 University of Pennsylvania School of Medicine, Philadelphia, USA

**Keywords:** Breast cancer, Sleep disturbance, Insomnia, Heart failure, Cardio-oncology, Left ventricular ejection fraction

## Abstract

Sleep disturbances during breast cancer (BC) treatment may represent a modifiable contributor to left ventricular ejection fraction (LVEF) decline during BC treatment. We investigated the association of sleep disturbance and LVEF decline in a prospective cohort of 247 women with non-metastatic BC scheduled for chemotherapy as part of the UPBEAT study conducted through the Wake Forest NCI Community Oncology Research Program Research Base (WF NCORP RB; WF-97415, UG1CA189824). Participants completed cardiac magnetic resonance imaging to measure LVEF, six-minute walk distance, and validated surveys assessing sleep disturbance, physical health, and mental health at baseline and 3 months. Sleep disturbance was measured using the Patient Reported Outcomes Measurement Information System Sleep Disturbance survey. At 3-month follow-up, 30.3% of participants experienced a clinically meaningful increase in sleep disturbance, with a mean LVEF decline of -4.13%-points compared to -1.93%-points in those without increased disturbance (*p* = 0.10). In multivariable models adjusting for baseline risk factors and concurrent changes in health, a 10-point increase in sleep disturbance was associated with a 1.31%-point reduction in LVEF (*β*= -0.131; 95% CI: -0.24,-0.02; *p* = 0.02). Associations were robust across sensitivity analyses. This study provides evidence linking sleep disturbance during BC treatment to early LVEF decline, suggesting sleep disturbance may contribute to subclinical cardiac dysfunction independent of general health decline. Longitudinal studies incorporating objective sleep measures are warranted to clarify mechanisms and evaluate whether behavioral interventions targeting sleep could mitigate cardiovascular risk in BC survivors.

## Background

Sleep disturbances – characterized by reduced sleep quality and/or duration – during breast cancer (BC) treatment represent an unexplored, potentially modifiable contributor to left ventricular ejection fraction (LVEF) decline [1], which is associated with significant morbidity and mortality [2]. Behavioral factors associated with treatment-related LVEF decline are important to characterize due to their potential for modifiability [3], and sleep is of particular concern given the American Heart Association’s inclusion of healthy sleep as one of the “Essential 8” measures of cardiovascular health [4]. Clinical and population-based studies have identified an association between sleep disturbance and incident cardiovascular disease [1], but this association has not been examined in women with BC. In a prospective cohort of women treated for BC with chemotherapy, our objective was to investigate the association between sleep disturbance during BC treatment and LVEF decline.

## Methods

Adult women with non-metastatic BC who were scheduled for chemotherapy treatment were recruited in the UPBEAT study (2038 approached, 247 enrolled [12.1%]), conducted through the Wake Forest NCI Community Oncology Research Program Research Base (WF NCORP RB; WF-97415, UG1CA189824; see Jordan et al. 2020 for full eligibility criteria) [5]. Enrolled between 7/21/2017 and 7/31/2021, participants completed cardiac magnetic resonance imaging (cMRI) to measure LVEF (typical range of 25%–75% with values below 60% indicating reduced LVEF for women), six minute walk distance (6MWD), and surveys to assess health status at baseline and 3-months after study enrollment. Surveys measured sleep disturbance (Patient Reported Outcomes Measurement Information System [PROMIS] Sleep Disturbance 8a normalized T-scores; range of 30.5–77.5 with scores ≥ 60 indicating clinically meaningful sleep disturbance), physical health (Short Form-36 [SF-36]), and mental health (SF-36 and Center for Epidemiologic Studies–Depression scale [CES-D]). The PROMIS Sleep Disturbance questionnaire measures the severity of problems with sleep quality, initiation, maintenance, and overall duration over the past week. Multiple readers assessed LVEF with discrepancies resolved by a study author (WGH). Missing data were imputed by multiple imputation with chained Eq [6]. The percentage missing per variable ranged from 1.2% to 14.6% across 15 variables, with 86 observations (34.8%) containing ≥ 1 missing value. Those with ≥ 1 missing value were more likely to be smokers (40.0% vs. 25.5%, *p* = 0.04) and were less likely to have received non-doxorubicin chemotherapy (85.1% vs. 74.4%, *p* = 0.04). There were no other significant differences at baseline.

The bivariate association of LVEF change with clinically meaningful sleep disturbance (T-score increase ≥ 10) was estimated with a T-test. Two multivariable linear models with bootstrapped confidence intervals were used to evaluate the association of the 3-month change sleep disturbance with change in LVEF while controlling for known risk factors for LVEF decline and for concurrent changes to mental and physical health. The initial model was adjusted for baseline values of sleep disturbance, LVEF, age, tobacco use (yes, no), BMI, racial classification (Black, White, and All Other Races: American Indian or Alaska Native, Asian, and Native Hawaiian or Pacific Islander), hypertension, high cholesterol, HER2 status, and BC treatment exposures (chemotherapy, radiation, and hormonal blockade). In the second model, both baseline and change values for physical health and mental health were included. A significance level of 0.05 was used for all statistical tests. All participants completed written informed consent, and ethics approval was obtained from the Wake Forest Institutional Review Board.

## Results

The sample (*N* = 247) mean age was 55.7 years (standard deviation [SD] = 10.9), with 19.0% Black participants and 75.7% White (Table 1). The mean LVEF at baseline was 60.7 (SD = 5.95) and the mean PROMIS Sleep Disturbance T-Score was 47.0 (SD = 9.91).

**Table 1:** Descriptive Statistics

	Total
	(*N* = 247)
Age (years)	55.7 (10.9)
Racial Classification	
Black	47 (19.0)
White	187 (75.7)
All Other Races^a^	13 (5.3)
Tobacco Use	63 (25.5)
Missing	31 (12.6)
Body Mass Index	29.4 (6.34)
Missing	3 (1.2)
Doxorubicin	117 (47.4)
Other Chemotherapy	201 (81.4)
Aromatase Inhibitors	114 (46.2)
Radiation	154 (62.3)
HER 2 Positive	59 (23.9)
Missing	7 (2.8)
Hypertension	68 (27.5)
Missing	32 (13.0)
High Cholesterol	73 (29.6)
Missing	33 (13.4)
Baseline LVEF	60.7 (5.95)
Missing	30 (12.1)
Six-Minute Walk Distance	465 (90.0)
Missing	3 (1.2)
SF-36 Physical Function	84.1 (19.5)
Missing	6 (2.4)
SF-36 Mental Health	74.1 (16.6)
Missing	6 (2.4)
CES-D Total	6.00 (4.77)
Missing	4 (1.6)
Baseline Sleep Disturbance T-Score	47.0 (9.91)
Missing	31 (12.6)

Continuous variables summarized with mean (standard deviation) and categorical variables summarized with count (percent)

*SF-36* Short Form 36, *LVEF* Left Ventricular Ejection Fraction, *CES-D* Center for Epidemiologic Studies - Depression

^a^Includes American Indian or Alaska Native, Asian, and Native Hawaiian or Pacific Islander

A total of 75 women (30.3%) experienced a meaningful increase in sleep disturbance, showing a mean 3-month LVEF change of −4.13% points (95% confidence interval [CI]: −1.76, −6.50) compared to a change of −1.93% points (95% CI: −0.80, −3.07) in the group with no clinically meaningful increase (*p* = 0.10). In the multivariable linear model, a 10-point sleep disturbance increase was associated with a 1.31%-point reduction in LVEF (*β*= −0.131; 95% CI: −0.24,−0.02; *p* = 0.02) (Table 2). In addition, baseline BMI, high cholesterol, HER2 positivity, and treatment with Doxorubicin and aromatase inhibitors were associated with LVEF declines, which is consistent with existing literature. Several sensitivity analyses did not alter the direction or statistical significance of the association.

**Table 2:** Multivariable Regression Results Examining the Changes in Sleep and Changes in Ejection Fraction in Women Undergoing Breast Cancer Treatment

	Model 1^a^			Model 2^b^		
	β	95% CI	*p*	β	95% CI	*p*
Age	0.084	0.01, 0.16	0.03	0.067	−0.01, 0.15	0.10
Racial Classification (ref: Black)						
White	−0.517	−2.72, 1.69	0.65	−0.128	−2.37, 2.12	0.91
All Other Races^c^	−2.805	−6.96, 1.35	0.19	−2.339	−6.37, 1.69	0.25
Tobacco Use	−1.495	−3.48, 0.49	0.14	−1.478	−3.60, 0.65	0.17
BMI	−0.132	−0.27, 0.00	0.06	−0.163	−0.31, −0.02	0.03
Doxorubicin	−3.58	−5.61, −1.56	< 0.001	−3.429	−5.54, −1.32	< 0.001
Other Chemotherapy	−1.746	−4.93, 1.44	0.28	−1.306	−4.60, 1.98	0.44
Aromatase Inhibitor	−2.08	−3.82, −0.34	0.02	−2.313	−4.11, −0.52	0.01
Radiation	1.921	−0.09, 3.93	0.06	1.584	−0.49, 3.65	0.13
HER2 Positive	−2.715	−5.02, −0.41	0.02	−2.677	−5.04, −0.31	0.03
Hypertension	0.405	−1.59, 2.40	0.69	0.298	−1.77, 2.36	0.78
High Cholesterol	−2.461	−4.48, −0.45	0.02	−2.141	−4.25, −0.04	0.05
Baseline LVEF	0.444	0.30, 0.59	< 0.001	0.42	0.27, 0.58	< 0.001
SF-36 Mental Health Change	–	–	–	0.003	−0.08, 0.08	0.94
SF-36 Phys. Function Change	–	–	–	0.039	−0.01, 0.08	0.08
6MWD Change	–	–	–	−0.006	−0.02, 0.01	0.38
CES-D Depression Change	–	–	–	0.097	−0.18, 0.37	0.49
PROMIS Sleep Disturbance Change	−0.118	−0.22, −0.01	0.03	−0.131	−0.24, −0.02	0.02

Confidence intervals estimated with bootstrapping. Parameters and p-values are pooled across 40 imputed datasets

*CI* Confidence Interval, *SF-36* Short Form 36, *6MWD* Six Minute Walk Distance, *LVEF* Left Ventricular Ejection Fraction, *PROMIS* Patient Reported Outcome Measure Information System, *CES-D* Center for Epidemiologic Studies - Depression

^a^ Result for PROMIS Sleep T-Score baseline adjustment parameter not shown

^b^ Results for the following baseline adjustment parameters are not shown: SF-36 Mental Health, SF-36 Physical Function, PROMIS Sleep T-Score, and CES-D

^c^ Includes American Indian or Alaska Native, Asian, and Native Hawaiian or Pacific Islander

## Discussion

To our knowledge, this is the first reported investigation connecting sleep disturbance during BC treatment to LVEF decline. While strengthened by prospective longitudinal data collection and precise quantification of LVEF with cMRI, the study is limited by the use of a self-reported sleep measure, and the direction of causation cannot be inferred since exposure and outcome were measured concurrently. The result was robust to adjustment for physical and mental health changes, suggesting a general decline in health does not fully account for the association. The magnitude of LVEF decline was substantial given the short follow-up time of only 3 months, particularly in those with a clinically meaningful increase in sleep disturbance. Though a decline of 5%-points is typically considered clinically meaningful, early subclinical declines in LVEF can portend further reductions as treatment progresses [7–9].

The association we report is consistent with several studies identifying insomnia as a risk factor for incident heart failure (HF), including a prospective sample of approximately 54,000 participants followed for over 11 years [1]. A separate prospective study of more than 80,000 post-menopausal women found insomnia to be a potent risk factor for cardiovascular disease [10], though in a sub-cohort of this population, who had incident BC during the study, no statistically significant association of insomnia with cardiovascular event risk was found [11]. However, this analysis considered insomnia as a binary variable and did not have precise measures of LVEF, which may explain the inconsistency with the current study.

The precise physiologic mechanism by which sleep disturbance could impact LVEF is unclear. Sleep-disordered breathing (e.g., sleep apnea) co-occurring with sleep disturbance could underly some of the observed association, as disordered breathing during sleep induces nighttime hypoxia, thoracic pressure variations, and left ventricular remodeling leading to LVEF decline or to HF with preserved LVEF [12]. Hyperarousal and sleep fragmentation associated with insomnia may engender increased sympathetic activity and inflammation during sleep time, leading to cardiovascular disease [1]. Additional study is needed to determine whether factors such as hyperarousal cause independent changes to both sleep and LVEF or whether sleep disturbance is causally implicated in in the development of Stage A HF or the transition from Stage A to Stages B or C. If causally related, behavioral intervention to treat sleep disturbance may be beneficial for mitigating LVEF decline to some degree. Objective measures of sleep, including polysomnography to identify sleep disordered breathing and actigraphy to measure changes to sleep latency, wake after sleep onset, and sleep duration would be beneficial for clarifying mechanisms in future studies.

These results suggest in-depth longitudinal investigation of sleep, HF, and BC treatment is warranted given cardiovascular disease remains a primary cause of early mortality among BC survivors. Characterizing sleep trajectories during and after BC treatment, ideally with both objective and subjective measures of sleep, may provide insights into associated cardiovascular effects, particularly the progression of HF in breast cancer patients from preclinical physiologic changes to symptomatic HF.

## Data Availability

The datasets generated and/or analysed during the current study are not publicly available to comply with data use agreements governing study data.

## Abbreviations

BC: Breast cancer
LVEF: Left ventricular ejection fraction
UPBEAT: Understanding and Predicting Breast Cancer Events After Treatment
NCORP: National Cancer Institute Community Oncology Research Program
PROMIS: Patient Reported Outcome Measures Information System
cMRI: Cardiac magnetic resonance imaging
BMI: Body mass index
HER2: Human epidermal growth factor receptor 2
SF-36: Short Form 36
6MWD: Six minute walk distance
CES-D: Center for Epidemiologic Studies – Depression
HF: Heart failure
